# Supplementary material for: Use of 3′ Rapid Amplification of cDNA Ends (3′ RACE)-Based Targeted RNA Sequencing for Profiling of Druggable Genetic Alterations in Urothelial Carcinomas
Source: Int J Mol Sci. 2024 Nov 12;25(22):12126. doi: 10.3390/ijms252212126 (PMC11594887; doi:10.3390/ijms252212126)
Supplement: Supplementary file 1 [file ijms-25-12126-s001.zip › Supplementary_Table_S5.pdf]

Supplementary Table S5. Gene names and underlying explanations.

Gene name	HGNC* approved full name	Previous name**
ADD1	adducin 1	adducin 1 (alpha)
BAIAP2L1	BAR/IMD domain containing adaptor protein 2 like 1	BAI1 associated protein 2 like 1
ARID1A	AT-rich interaction domain 1A	SWI/SNF related, matrix associated, actin dependent regulator of chromatin, subfamily f, member 1; AT rich interactive domain 1A (SWI-like); AT rich interactive domain 1A (SWI-like)
BICC1	BicC family RNA binding protein 1	bicaudal C homolog 1 (Drosophila)
BRAF	B-Raf proto-oncogene, serine/threonine kinase	v-raf murine sarcoma viral oncogene homolog B
CASP7	caspase 7	caspase 7, apoptosis-related cysteine protease;<br>caspase 7, apoptosis-related cysteine peptidase
DDX23	DEAD-box helicase 23	PRP28 homolog, yeast;<br>DEAD (Asp-Glu-Ala-Asp) box polypeptide 23
CCND1	cyclin D1	cyclin D1 (PRAD1: parathyroid adenomatosis 1)
CD274 (PD-L1)	CD274 molecule	Programmed cell death 1 ligand 1
CDKN2A	cyclin dependent kinase inhibitor 2A	cyclin-dependent kinase inhibitor 2A (melanoma, p16, inhibits CDK4)
CDKN2B	cyclin dependent kinase inhibitor 2B	cyclin-dependent kinase inhibitor 2B (p15, inhibits CDK4)
ERBB2 (HER2)	erb-b2 receptor tyrosine kinase 2	v-erb-b2 avian erythroblastic leukemia viral oncogene homolog 2 (neuro/glioblastoma derived oncogene homolog);<br>v-erb-b2 avian erythroblastic leukemia viral oncogene homolog 2
FGFR1	fibroblast growth factor receptor 1	fms-related tyrosine kinase 2
FGFR2	fibroblast growth factor receptor 2	bacteria-expressed kinase;<br>keratinocyte growth factor receptor;<br>craniofacial dysostosis 1;<br>Jackson-Weiss syndrome
FGFR3	fibroblast growth factor receptor 3	achondroplasia, thanatophoric dwarfism
FGFR4	fibroblast growth factor receptor 4	
GOLGA5	golgin A5	golgi autoantigen, golgin subfamily a, 5
HRAS	HRas proto-oncogene, GTPase	v-Ha-ras Harvey rat sarcoma viral oncogene homolog;<br>Harvey rat sarcoma viral oncogene homolog
KDM6A	lysine demethylase 6A	ubiquitously transcribed tetratricopeptide repeat, X chromosome
KMT2D	lysine methyltransferase 2D	trinucleotide repeat containing 21,<br>myeloid/lymphoid or mixed-lineage leukemia 2,<br>lysine (K)-specific methyltransferase 2D
KRAS	KRAS proto-oncogene, GTPase	v-Ki-ras2 Kirsten rat sarcoma 2 viral oncogene homolog
MDM2	MDM2 proto-oncogene	mouse double minute 2, human homolog of p53-binding protein; Mdm2, transformed 3T3 cell double minute 2, p53 binding protein (mouse); Mdm2 p53 binding protein homolog (mouse); MDM2 proto-

		oncogene, E3 ubiquitin protein ligase
NRAS	NRAS proto-oncogene, GTPase	neuroblastoma RAS viral (v-ras) oncogene homolog
PIK3CA	phosphatidylinositol-4,5-bisphosphate 3-kinase catalytic subunit alpha	phosphoinositide-3-kinase, catalytic, alpha polypeptide;<br>phosphatidylinositol-4,5-bisphosphate 3-kinase, catalytic subunit alpha
RB1	RB transcriptional corepressor 1	Osteosarcoma; retinoblastoma 1
SEL1L	SEL1L adaptor subunit of SYVN1 ubiquitin ligase	sel-1 suppressor of lin-12-like (C. elegans);<br>SEL1L, ERAD E3 ligase adaptor subunit;<br>SEL1L adaptor subunit of ERAD E3 ubiquitin ligase
SMIM14	small integral membrane protein 14	chromosome 4 open reading frame 34
TACC3	transforming acidic coiled-coil containing protein 3	transforming, acidic coiled-coil containing protein 3
TERT	telomerase reverse transcriptase	
TP53	tumor protein p53	
UACA	uveal autoantigen with coiled-coil domains and ankyrin repeats	

* HUGO Gene Nomenclature Committee at the University of Cambridge

** Gene names often reflect rather historical context of their discovery than actual gene function or its medical significance
